# Bone Mass and Sexual Dimorphism in Clarke’s Angle: A Multivariate Regression Approach to the Medial Longitudinal Arch in University Students

**DOI:** 10.3390/jfmk11020230

**Published:** 2026-06-06

**Authors:** Donalds Steven Guali, Victor Manuel Piamba Ome, Armando Monterrosa-Quintero, Boryi A. Becerra-Patiño, Luis Gabriel Rangel Caballero, Adrián De la Rosa

**Affiliations:** 1 Physical Education and Sports Program, Altius Performance Laboratory, Universidad Surcolombiana, Neiva 410001, Colombia; u20201185482@usco.edu.co (D.S.G.); u20201185488@usco.edu.co (V.M.P.O.); 2 Faculty of Physical Education, National Pedagogical University, Bogotá 111166, Colombia; babecerrap@pedagogica.edu.co; 3 Grupo Ser, Cultura y Movimiento, Facultad de Cultura Física Deporte y Recreación, Universidad Santo Tomás, Bucaramanga 681011, Colombia; dcultu@ustabuca.edu.co; 4 Body, Physical Activity and Sport Study Group (GECAFD), Sports Department, Universidad Industrial de Santander, Bucaramanga 680002, Colombia; amdegon@uis.edu.co; 5 Freshage Research Group, Department of Physiology, Faculty of Medicine, University of Valencia, CIBERFES, Fundación Investigación Hospital Clínico Universitario/INCLIVA, 46010 Valencia, Spain

**Keywords:** plantar footprint, Clarke’s Angle, bone mass, sexual dimorphism, digital photogrammetry, Elastic Net

## Abstract

**Background:** Flattening of the medial longitudinal arch is traditionally attributed to excess body weight and Body Mass Index (BMI). However, controversy exists regarding whether adiposity or skeletal structure drives this biomechanical alteration, and which podometric index best detects it. **Methods:** A cross-sectional study evaluated 99 healthy university students (50 males, 49 females). Body composition was assessed via a four-component model. Plantar footprints were captured using 4K digital podoscopy and analyzed with five morphometric indices. Arch predictors were identified using multivariate regression models (Elastic Net regression) and Generalized Additive Models (GAMs). **Results:** Only Clarke’s Angle detected significant sexual dimorphism, showing structurally higher arches in females (50.28° ± 7.14) than in males (41.82° ± 11.20; *p* < 0.001). Multivariate analysis revealed bone mass as the dominant structural predictor, exerting a non-linear negative association with the arch profile, which stabilizes beyond 12 kg. BMI was not a significant predictor, whereas body fat percentage showed a modest positive association. **Conclusions:** Plantar arch morphology is strongly associated with skeletal load (anthropometrically estimated bone mass) rather than adiposity or BMI. Within this specific cohort, Clarke’s Angle emerged as a highly sensitive instrument for characterizing sexual dimorphism. Clinical assessments diagnosing functional flatfoot should prioritize underlying bone structure over BMI, particularly when evaluating a healthy and physically active university population. Future studies incorporating DXA or radiographic validation are needed to confirm these anthropometric findings.

## 1. Introduction

The architecture of the human foot, particularly the integrity of the Medial Longitudinal Arch (MLA), is a cornerstone of biomechanical efficiency in bipedal locomotion and dynamic postural stability [1]. The MLA serves as the body’s primary shock absorber and load distributor. Consequently, morphological variations, whether through flattening (pes planus) or excessive elevation (pes cavus), have been consistently linked to altered plantar pressure distribution, impaired static balance, and a heightened risk of lower-limb musculoskeletal injuries [2,3].

Historically, radiography has been the gold standard for MLA assessment [4,5]. However, concerns about ionizing radiation exposure and the logistical demands of clinical settings have paved the way for photogrammetry and digital podometry [6,7]. These techniques have emerged as reliable, non-invasive, and cost-effective alternatives for large-scale clinical screening [6]. While cutting-edge technologies such as electronic baropodometry and finite element analysis provide high-fidelity data on dynamic loading and functional morphology [8,9], their integration into large-scale population studies is often hindered by technical complexity and prohibitive operational costs [10].

Despite the ubiquity of footprint analysis, a significant gap persists in the literature regarding the diagnostic agreement among the various geometric indices available [11]. Recent research suggests that traditional metrics such as the Chippaux–Smirak index, the Staheli index, and Clarke’s Angle are not interchangeable and exhibit varying diagnostic sensitivity [12]. Specifically, there is ongoing debate about their ability to detect subtle variations associated with sexual dimorphism [5]. While some recent studies report no significant links between gender and arch height [13], other authors argue that intrinsic biomechanical differences may be overlooked if the wrong morphometric index is chosen, pointing to Clarke’s Angle as a potentially more discriminative metric in certain cohorts [2,14].

A critical point of contention concerns the anthropometric determinants of foot posture [15]. Classical literature has long established a positive correlation between excess body weight and arch collapse, linking obesity to a higher prevalence of flatfoot [13,14]. Nevertheless, the body mass index (BMI) remains a blunt instrument; it fails to distinguish between the mechanical loads imposed by adiposity, muscle mass, or underlying skeletal structure [8]. Emerging evidence suggests that fractional body composition may explain far more of the variance in foot morphology than total weight alone [16]. This raises the hypothesis that specific structural components, such as bone mass, play a decisive role, which has been largely ignored by the simple linear regression models traditionally employed [17,18].

Accordingly, the objective of this study was twofold: (1) to compare the diagnostic sensitivity of five validated podometric indices in characterizing sexual dimorphism among university students, testing the hypothesis that Clarke’s Angle offers superior discriminatory power over width-ratio indices; and (2) to determine the influence of fractional body composition (fat, muscle, and bone mass) on arch structure using robust multivariate frameworks, specifically Elastic Net and Generalized Additive Models (GAMs).

## 2. Materials and Methods

### 2.1. Study Design and Ethical Considerations

A descriptive, correlational, and comparative cross-sectional study was conducted among university students at the Universidad Surcolombiana. The research protocol strictly adhered to the Declaration of Helsinki and was governed by Resolution 8430 of 1993, issued by the Colombian Ministry of Health, which defines the ethical and technical guidelines for health research. All athletes included provided written informed consent for testing and data collection. The research complied with the Helsinki Declaration, and the protocol was approved by the Ethics Committee for Human Beings at the Universidad Santo Tomás (Approval No. 06062025; 6 June 2025). All procedures were validated and supervised by the ALTIUS Laboratory for Physical Performance Evaluation and Development.

### 2.2. Participants and Sampling Strategy

The sample comprised 99 healthy volunteers, including 50 men (age: 21.7 ± 2.96 years; height: 174 ± 6.46 cm; body mass: 72 ± 10.3 kg; BMI: 23.7 ± 2.65 kg/m^2^) and 49 women (age: 21.7 ± 2.85 years; height: 160 ± 6.69 cm; body mass: 59.4 ± 9.32 kg; BMI: 27.4 ± 3.06 kg/m^2^). Sampling was stratified by age and sex and adjusted for a finite population at the 80% confidence level. Inclusion required participants to be active students enrolled in the Physical Education, Recreation, and Sports program, whereas exclusion criteria focused on amputations or lower-limb injuries that could impair accurate evaluation and biomechanical analysis of the plantar footprint.

### 2.3. Experimental Protocol and Anthropometric Assessment

Participants were assessed in standardized sports attire (lycra clothing for men; lycra and sports tops for women) during morning sessions to control for circadian variance. To ensure data integrity, subjects were required to be in a rested state and to avoid physical activity or significant perspiration prior to evaluation. The protocol followed a two-phase sequence:

1. Plantar Data Acquisition: Subjects were positioned in a neutral orthostatic posture (gaze forward, arms adducted) on a digital podoscope. Images were captured only after postural stabilization was confirmed. Digital footprints were processed in CorelDRAW Graphics Suite 2024 (version 25.0; Corel Corporation, Ottawa, Canada) to calculate and classify morphometric indices using the authors’ specific scales.
2. Morphological Evaluation: Body mass was measured with a calibrated Ironman Series digital scale (Tanita Corp., Tokyo, Japan) (100 g precision), and height was recorded with a Seca 206; Seca GmbH & Co. KG, Hamburg, Germany (0.1 cm precision). Skinfolds (triceps, subscapular, iliac crest, and abdominal), circumferences (waist and hip), and bone diameters (bi-styloid and femoral) were measured in triplicate using high-precision Cescorf Equipamentos Esportivos Ltda, Porto Alegre, Brazil (skinfold caliper: 10 g/mm^2^ constant pressure; flexible steel tape; and 60 cm anthropometer).

Body composition was estimated using a four-component model. Body fat percentage was determined using Faulkner’s protocol, which is based on the cumulative thickness of four skinfolds (triceps, subscapular, iliac crest, and abdominal) [19]. Bone mass was calculated from the bicondylar breadths of the humerus and femur [20]. Residual mass was calculated as a fixed proportion of body mass for males and females [21]. Finally, muscle mass was indirectly estimated by subtracting the sum of fat mass, bone mass, and residual mass from total body mass.

### 2.4. Data Collection, Protocols, and Instruments

All anthropometric measurements were performed during morning sessions to minimize circadian variability and ensure data reliability. A comprehensive morphofunctional profile was recorded, including sociodemographic and anthropometric variables such as sex, age, body mass, height, and BMI. The morphological assessment included the waist-to-hip ratio (WHR), bone diameters (bistyloid and femur), and skinfold thickness (triceps, subscapular, iliac crest, and abdominal). The core analysis involved bilateral plantar footprints, and morphometric indices were calculated using methodologies from five validated authors: Chippaux–Smirak, Staheli, Clarke, the Arch Index, and Valenti, providing a multidimensional characterization of the medial longitudinal arch.

#### 2.4.1. Instrumentation and Digital Image Capture

**Figure 1:**
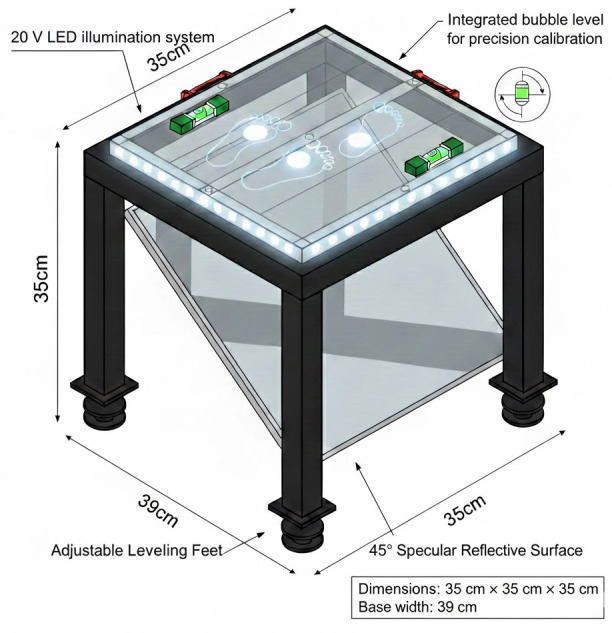
Digital podoscope set up for bilateral plantar footprint acquisition.

Plantar footprint morphology was assessed using a custom-engineered digital capture podoscope (Figure 1). The device structure consisted of a leveled steel frame equipped with four screw-type surface levelers to ensure structural stability. The system integrated a tempered-glass platform and a high-reflectivity mirror inclined at 45°. To ensure a high-contrast visualization of the pressure distribution, the perimeter was illuminated with high-intensity LED lighting. Prior to each session, the apparatus was calibrated using high-precision spirit levels to eliminate any slope-related measurement bias and ensure a perfectly horizontal assessment surface, as illustrated in Figure 1.

Digital images were acquired using an EMEET SmartCam C960 4K (SHENZHEN EMEET TECHNOLOGY Co., Ltd., Shenzhen, China), featuring a high-resolution Sony sensor and Phase Detection Autofocus (PDAF) for stable acquisition. To ensure spatial accuracy, a physical metric reference scale was placed on the glass surface during each capture, enabling precise pixel-to-millimeter calibration in CorelDraw 2024 (Corel Corp., Ottawa, ON, Canada). This procedure ensured that all subsequent morphometric digitizing and linear measurements maintained a high degree of geometrical fidelity.

#### 2.4.2. Plantar Footprint Morphometric Analysis

**Figure 2:**
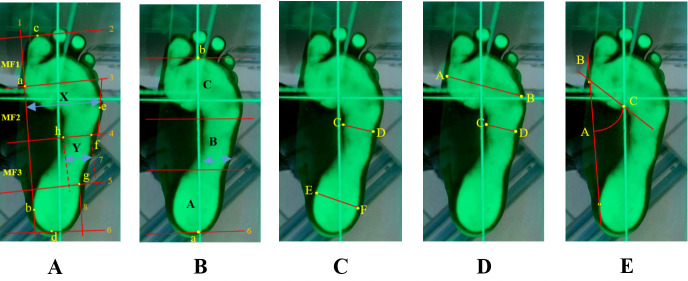
Graphic representation of the morphogeometric indices and measurement protocols used for footprint analysis. (**A**) Hernández-Corvo Index (IHC): Protocol based on the identification of anatomical points a–h and the tracing of fundamental measurement lines (MF1–MF3), where horizontal lines 1–6 delimit the footprint's longitudinal zones, and segments X and Y represent the maximum metatarsal and minimum arch widths respectively. (**B**) Arch Index (IA): The footprint is divided into three equal longitudinal areas hindfoot (A), mediopié (B), and antepié (C) based on a central axis from the midpoint of the heel (point a) to the second toe, with point b marking the tangential intersection. (**C**) Staheli Index (SI): This index establishes the ratio between the minimum transverse width of the midfoot (segment CD) and the maximum width of the heel or hindfoot region (segment EF). (**D**) Chippaux–Smirak Index (CSI): Calculation of the ratio between the minimum midfoot width (segment CD) and the maximum metatarsal or forefoot width (segment AB). (**E**) Clarke’s Angle (AA): Measurement of the medial arch concavity angle, where line B connects the medial metatarsal edge with the peak of the longitudinal arch at point C, and the vertical reference line represents the tangential medial border.

The bilateral plantar footprints were analyzed using five validated morphogeometric methods to ensure a multidimensional characterization of the MLA (Figure 2). To ensure measurement consistency and mitigate inter-rater variability, all morphometric digitizations and categorizations were meticulously performed by a single experienced researcher. While formal intra-rater reliability coefficients were not specifically quantified for this cohort, the geometric protocols utilized have demonstrated high reproducibility in previous clinical literature.

#### 2.4.3. Hernández-Corvo Index (HCI)

Based on the protocol by [22], this method quantifies the relationship between the midfoot (y) and the forefoot (x) widths. Following the identification of anatomical landmarks (a–h) and the fundamental measurements (FM), the index was calculated using the equation: HCI = (y/x) × 100 [23].

#### 2.4.4. Arch Index (AI)—Cavanagh & Rodgers

According to Cavanagh and Rodgers [24], the footprint (excluding toes) was divided into three equal regions (forefoot, midfoot, and rearfoot) along a central axis. The AI was defined as the ratio of the midfoot area (B) to the total footprint area (A + B + C): AI = B/(A + B + C).

#### 2.4.5. Staheli Index (SI)

This index measures the ratio of the minimum midfoot width (CD) to the maximum heel width (EF). The classification followed the criteria of Staheli et al. [25], in which values between 0.6 and 0.7 indicate a normal arch.

Chippaux–Smirak Index (CSI)*:* As described by [26], the CSI expresses the ratio between the narrowest part of the midfoot and the widest part of the forefoot. The classification thresholds identify cavus (<0.1%), normal (0.1–29.9%), intermediate flat foot (30.0–44.9%), and severe flat foot (≥45%).

#### 2.4.6. Clarke’s Angle (α)/Alpha Angle

Based on [27], this geometric construction measures the medial concavity. It is formed by the line connecting the most medial points of the forefoot and heel, and a second line tangential to the peak of the medial arch concavity.

### 2.5. Statistical Analysis

Data analysis was performed using R Statistical Software (version 4.5.2). The distributional properties of the variables were assessed for normality using the Kolmogorov–Smirnov test, and homoscedasticity was verified using Levene’s test. Descriptive statistics are presented as mean ± standard deviation (SD) for parametric data and as medians with Interquartile Range (IQR) values for non-parametric variables.

Bivariate comparisons between sexes were conducted using the Mann–Whitney U test for non-normally distributed data and a one-way ANOVA for parametric comparisons. To explore associations between body composition markers and plantar indices, Spearman’s rank correlation matrices were constructed. Categorical data were analyzed using Chi-squared (χ^2^) tests. To mitigate multicollinearity and perform robust variable selection, a Penalized Regression model (Elastic Net) was implemented. Finally, GAMs were used to capture and visualize nonlinear dependencies between anthropometric predictors and plantar morphology, thereby ensuring a high-fidelity interpretation of biomechanical interactions. To estimate the effect size between groups, epsilon squared was employed, following the thresholds proposed by Cohen [28] and Tomczak & Tomczak [29]; these were categorized as small (0.01 to <0.08), medium (0.08 to <0.26), or large (≥0.26).

## 3. Results

**Figure 3:**
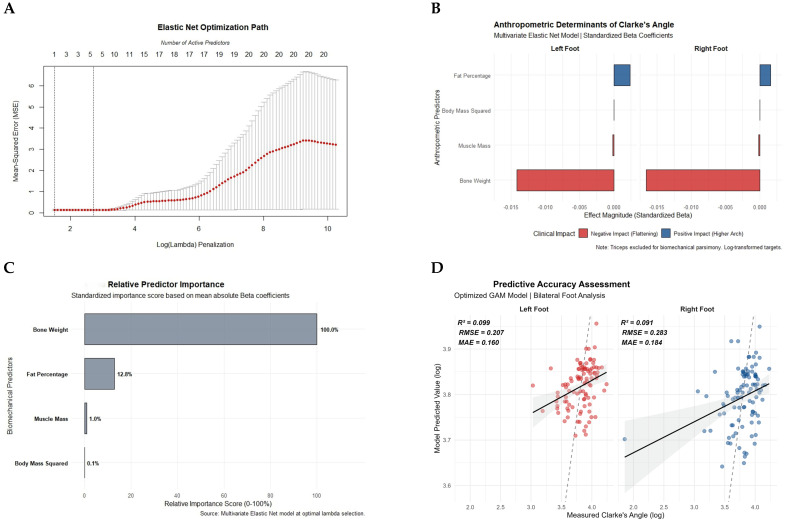
Multivariate Elastic Net regression framework for the prediction of Clarke’s Angle based on body composition determinants. (**A**) Cross-validation optimization path for the penalization parameter (λ); vertical dashed lines indicate the minimum MSE (λ_min_) and the 1-standard-error rule (λ_1se_) used for model parsimony. (**B**) Standardized Beta coefficients for the selected structural predictors, showing the consistent negative effect of Bone Weight (red) and the positive effect of Fat Percentage (blue) on arch height. (**C**) Global relative variable importance, demonstrating that skeletal mass (100%) outweighs fat percentage (12.8%) and muscle mass (1.0%) as the primary driver of variance. (**D**) Model performance metrics comparing log-transformed observed and predicted values for bilateral footprints, indicating goodness of fit (R^2^ ≈ 0.09−0.10) and predictive accuracy.

**Figure 4:**
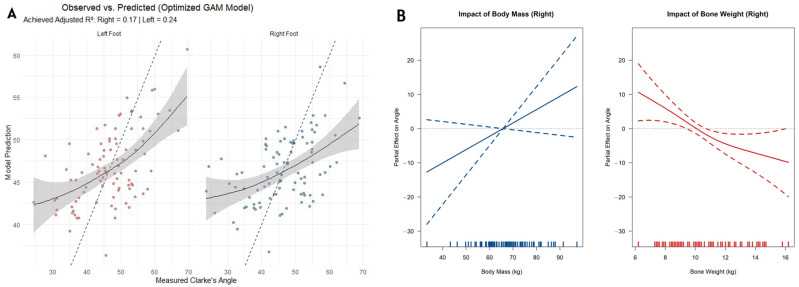
Comprehensive Diagnostic of the Optimized Multivariate GAM Model. (**A**) Scatterplots for both Left and right feet illustrate the achieved predictive accuracy, with adjusted R^2^ values of 0.24 and 0.17, respectively. The alignment between measured and predicted Clarke’s Angles indicates that the model successfully captures the structural variance of the foot arch, even within the complex biological noise inherent in biometric data. (**B**) Partial effect plots reveal the distinct mechanical drivers of the arch angle. Body Mass shows a positive linear correlation, with higher mass associated with a higher predicted angle. In contrast, Bone Weight exhibits a pronounced non-linear negative effect, identifying it as the critical structural determinant: as bone mass increases, the arch angle significantly decreases (flattening effect), reaching a stabilization point beyond 12 kg. Shaded areas and dashed lines represent 95% confidence intervals, confirming the statistical stability of the estimates across the predictor range.

This section presents the main findings of the plantar footprint assessment conducted in a population of university students. Table 1 reports the descriptive statistics of anthropometric and body composition variables stratified by sex. Table 2 presents a comparison between males and females using the different protocols applied to determine plantar footprints for both the right and left feet. Table 3 shows the relationships between anthropometric and body composition variables and the indices used to assess the plantar footprint, whereas Table 4 presents the association between sex and the plantar footprint index, which was statistically significant. Additionally, multivariate regression analyses are presented in Figure 3 and Figure 4, which allowed for the identification of the predictors influencing plantar arch measurements, as determined by the Clarke’s Angle.

The study sample comprised a homogeneous cohort of university students, with a median age of 22 years (range: 17–30) in both sexes (*p* = 0.602). Descriptive analyses revealed marked sexual dimorphisms in body composition and structural parameters (Table 1). Male participants had significantly higher absolute body mass and height than female participants (*p* < 0.001). These structural differences were further supported by pronounced skeletal and muscular contrasts, with males presenting substantially greater bone mass (ε^2^ = 0.64) and muscle mass (ε^2^ = 0.31). Conversely, females exhibited a distinct adiposity pattern, characterized by significantly higher triceps and iliac crest skinfold measurements (*p* < 0.001, ε^2^ = 0.30), typical of this biological group. Despite these sex-related differences, BMI did not differ significantly between groups (*p* = 0.464), indicating that the observed variations in body mass are largely attributable to differences in tissue distribution rather than overall body size relative to height. Additionally, males had a significantly higher WHR (0.83 vs. 0.77; ε^2^ = 0.48), consistent with the expected android and gynoid fat distribution patterns in this young adult population.

The morphofunctional analysis of the plantar footprint showed high bilateral symmetry across all assessed indices in both sexes (Table 2). According to the Hernández-Corvo classification, participants in both groups exhibited values corresponding to a normal-to-moderately high arch profile, with no statistically significant differences between the right and left feet (e.g., females: right 65.01 ± 9.30 vs. left 64.93 ± 7.02). In addition, the Cavanagh AI and the HCI showed consistent values between males and females, indicating that the midfoot contact area remains proportional to overall foot dimensions in this young adult population. A notable difference was observed in Clarke’s Angle, with female students presenting significantly higher values (50.28 ± 7.14°) than male students (41.82 ± 11.20°). This result, together with the Staheli Index values (ranging from 57.86 to 63.42), suggests that although total plantar contact area is comparable between sexes, the structural height of the medial longitudinal arch tends to be more pronounced in females. Overall, these findings indicate that despite the greater body mass previously observed in males, plantar load distribution remains functionally balanced in both groups within the university athletic context.

Table 3 shows that the associations with the greatest practical magnitude, according to Hopkins’ criteria, are mainly concentrated in Clarke’s Angle, particularly for the left foot. For this index, the largest coefficients are observed with variables related to bone structure, with bone mass and femur breadth standing out as the strongest associations in the analysis. Similarly, for the right foot, Clarke’s Angle, the most relevant associations are again linked to skeletal variables, especially bone mass and femur breadth, exceeding those observed with overall body mass and soft-tissue distribution variables in magnitude. In contrast, the left-foot Cavanagh Arch Index shows associations of lower magnitude, mainly in the trivial-to-small range, and therefore does not reach the levels of practical relevance observed in Clarke’s Angle. Overall, under the Hopkins framework, the findings indicate that the largest and most practically meaningful coefficients are consistently associated with bone-related indicators, highlighting Clarke’s Angle as the most sensitive foot index for capturing variations related to skeletal characteristics.

A significant association between sex and foot type was observed exclusively for the plantar morphology assessed by the Clarke’s Angle method in both the right (χ^2^ =24.10, df = 4, *p* < 0.001) and left feet (χ^2^ =28.92, df = 4, *p* < 0.001), (Table 4). These findings were further corroborated by Fisher’s Exact Test (*p* < 0.001), ensuring the validity of the results despite low frequency counts in certain categories. The strength of this association was substantial, as indicated by a Cramer’s V of 0.49 for the right foot and 0.54 for the left foot, reflecting a strong sex-dependent effect on foot architecture.

Detailed analysis of adjusted residuals revealed distinct sexual dimorphism: females exhibited a significantly higher prevalence of pes cavus (71% in the right foot) and normal foot types. Conversely, males were predominantly characterized by a collapse or flattening of the arch, showing significantly higher frequencies of intermediate, flat, and plano-normal feet. Notably, flatfoot emerged as a condition exclusive to males in this sample (z = −2.44 for females). These results suggest that Clarke’s Angle is a highly sensitive metric for capturing sex-related differences in foot morphology, whereas other plantar indices failed to detect such significant variations.

### Multivariate Modeling of Anthropometric Predictors

To identify the structural predictors of the medial longitudinal arch, a penalized regression model (Elastic Net) was optimized using cross-validation. As shown in Figure 3A, the optimal penalization parameter (λ_min_) minimized mean squared error while retaining key predictors, whereas the parsimonious model (λ_1se_) achieved a substantial reduction in complexity without a statistically significant loss in predictive power. The analysis revealed a consistent biomechanical pattern across both feet (Figure 3B). Bone Weight emerged as the most significant structural predictor, exhibiting a strong negative association with Clarke’s Angle. This indicates that individuals with higher skeletal mass tend to have lower arch angles (flatter arches), independent of adiposity. In contrast, Body Fat Percentage showed a modest positive association with the arch angle, whereas Muscle Mass showed a negligible effect, being nearly neutralized by the regularization penalty.

The hierarchy of predictor importance confirmed these findings (Figure 3C). Bone Weight achieved the highest relative importance score among the evaluated variables, emerging as the primary statistical predictor of Clarke’s Angle within this cohort. Secondary factors, such as Fat Percentage (12.8%) and Muscle Mass (1.0%), contributed significantly less to the model. Finally, the goodness-of-fit assessment (Figure 3D) yielded R^2^ values of 0.099 and 0.091 for the left and right foot, respectively. While the model captures the significant linear trend driven by skeletal structure, the scatterplots suggest that a portion of arch variability remains influenced by nonlinear factors or variables not included in the standard anthropometric profile.

Figure 4 shows partial effect plots derived from the GAM, depicting the non-linear effects of body mass and bone weight on Clarke’s angle for the right foot.

## 4. Discussion

A key finding of this study is the identification of bone mass, rather than BMI or adiposity, as a highly significant structural factor influencing MLA height. However, it must be acknowledged that our predictive models captured a modest portion of the overall variance (R^2^ ≈ 0.09–0.10). This indicates that while skeletal weight is the strongest anthropometric predictor among those evaluated, foot arch morphology is profoundly multifactorial. A substantial portion of the unmeasured variance is likely governed by alternative biomechanical drivers, such as intrinsic ligamentous laxity, plantar fascia stiffness, and active neuromuscular control (e.g., the foot core system) [30]. While classical literature has consistently established an inverse linear relationship between excess weight and arch integrity, attributing flatfoot to the mechanical load imposed by obesity [3,13,14,31], our multivariate models (Elastic Net and GAM) challenge this simplification. By fractionating body composition, we found that skeletal weight exhibits the highest relative importance among the evaluated anthropometric variables, showing a non-linear negative association that stabilizes at bone mass above 12 kg. This suggests that arch flattening in healthy, active young adults is not necessarily a consequence of “fatness” [32] or of ligamentous laxity but rather hypothesized as a biomechanical adaptation to a heavier, more robust skeletal structure [33]. This result supports the hypothesis of Monterrosa-Quintero et al. (2023) [8] that BMI is insufficient to describe functional morphology in athletic populations, in which elevated body weight often derives from lean tissue (bone and muscle) rather than adipose tissue. However, it is crucial to clarify for clinical interpretation that our findings do not imply that obesity is not a risk factor for flatfoot. In the general population, particularly among sedentary or clinically obese individuals, the mechanical overload of excessive adipose tissue likely plays a fundamentally different and deleterious role that cannot be directly extrapolated from the cohort of university students evaluated in the present study.

Regarding the controversy over the interchangeability of podometric indices, our results confirmed that not all methods have the same diagnostic sensitivity, consistent with recent reports [12]. One of the most critical findings was that among the five indices evaluated, only Clarke’s Angle detected significant sexual dimorphism, revealing that women have higher arches (~50°) than men (~42°). Conversely, indices based on width ratios (Chippaux–Smirak, Staheli, and Arch Index) found no sex differences [34]. This could explain why previous studies, such as Saadah et al. [13], reported no gender differences; it is likely that the choice of “contact area” indices (which depend on the plantar fat pad) masks real architectural differences in the arch that only an angular index, such as Clarke’s, is able to capture [35]. Therefore, Clarke’s Angle is proposed as a highly reliable measure for investigating sexual dimorphism and variations in the medial longitudinal arch through footprint evaluation, offering greater sensitivity than other footprint indices within this specific university student population.

The observed sexual dimorphism, in which women had a higher prevalence of cavus and normal feet despite significantly greater adiposity (significantly higher skinfolds), supports our hypothesis regarding skeletal load [36]. Men in our sample had significantly higher bone mass (*p* < 0.001, ε^2^ = 0.64), which, according to our regression model, is the factor that “flattens” the arch. This contradicts the popular notion that body fat is the primary factor in arch collapse in youth; in fact, our Elastic Net model showed that fat percentage had a modest positive association with the arch angle. This indicates that, in the absence of morbid obesity, adipose tissue might not be mechanically deleterious to the arch in young women, whereas the static load of a heavy male skeleton imposes greater demand on the foot’s passive subsystem (fascia and ligaments), resulting in a flatter profile [37,38].

From a clinical and methodological perspective, this study validates the use of low-cost digital podometry as a robust alternative to radiography and expensive baropodometry [6,10]. Implementing a 4K camera with pixel-to-millimeter calibration provided sufficient precision to feed our multivariate frameworks. The practical implication is direct: clinicians and epidemiologists should be cautious when diagnosing arch pathology based solely on BMI. An individual with a high BMI due to muscular hypertrophy and increased bone density (common in physical education students and athletes) may present with a lower arch (reduced Clarke’s Angle) as a stable functional adaptation, not necessarily as a “pathological flatfoot” requiring orthopedic intervention [27].

### Limitations and Future Directions

Although this study provides robust multivariate evidence regarding the determinants of the medial longitudinal arch, certain methodological and instrumental limitations must be acknowledged. First, the sample consisted exclusively of university students from a Physical Education and Sports program. This population is characterized by high levels of physical activity and muscle density, which limits the generalizability of our findings to sedentary populations, the elderly, or individuals with metabolic obesity, where the impact of adipose tissue might differ from the hypothesized skeletal loading associations observed here. Therefore, a clear distinction must be made: while our results demonstrate the primary role of skeletal load in active individuals, they do not negate the well-established risk that excessive adiposity poses to arch integrity in obese populations.

Second, regarding anatomical and structural validation, this study relied on superficial photogrammetry and indirect anthropometry.

A core limitation was the absence of radiological evaluation, which has historically been considered the gold standard for assessing actual bone alignment (e.g., the talometatarsal angle). Similarly, while a four-component model was utilized for body composition, Dual-Energy X-ray Absorptiometry (DXA) was not employed to quantify bone mineral density with clinical precision. Future research should correlate our photogrammetric findings with radiographic imaging to confirm whether the anthropometrically estimated “bone mass” corresponds to a more rigid skeletal architecture.

Third, the morphometric analysis was limited to static plantar footprints obtained via a digital podoscope. The study did not include electronic baropodometry platforms, thereby precluding analysis of dynamic plantar pressure distributions. While static evaluation is valid for screening, it does not fully capture the foot’s functional behavior during the gait cycle. It is imperative that future research integrate baropodometric analyses to determine whether the structural rigidity associated with a low Clarke’s Angle (high bone mass) generates pathological pressure peaks or kinematic alterations during locomotion. Theoretically, an increased skeletal mass may confer greater structural stiffness to the foot. This could act as a biomechanical compensatory mechanism during the gait cycle, helping to maintain propulsion efficiency and dynamic stability despite presenting a structurally lower or flattened arch profile under static conditions.

## 5. Conclusions

This study provides robust multivariate evidence that suggests bone mass, rather than adiposity or BMI, as the primary anthropometric predictor of the medial longitudinal arch height in healthy young adults. Contrary to the traditional assumption that excess body weight universally flattens the foot, our GAMs and Elastic Net analyses revealed that a heavier skeletal structure exerts a non-linear negative effect on the arch, stabilizing beyond a critical bone mass threshold of 12 kg. This suggests that in active populations, arch morphology is mainly associated with osteogenic adaptation to static load rather than the mechanical burden of adipose tissue.

Methodologically, within the studied population, Clarke’s Angle proved to be a highly sensitive index, as it was the only measure capable of detecting sexual dimorphism not captured by width-based indices such as Chippaux–Smirak and Staheli. Females showed higher structural arches despite greater skinfold thickness, supporting the notion that contact area-based indices may be influenced by the plantar fat pad rather than by true skeletal structure.

Clinically, the results support the use of high-resolution digital photogrammetry as a valid and cost-effective screening method. Flatfoot risk assessment should not rely solely on BMI, especially in athletic populations, because a lower arch in individuals with high bone mass may reflect a stable functional adaptation rather than pathology. Future research should incorporate dynamic gait analysis to better understand how these structural factors influence locomotor mechanics.

## Figures and Tables

**Table 1:** Descriptive and comparative characteristics by sex, expressed as Median and IQR.

Variable	Female	Male	*p*-Value	ε^2^
Body mass (kg)	60.80 (5.75)	70.10 (14.67)	<0.001	0.33
Height (cm)	158.95 (7.25)	174.45 (8.05)	<0.001	0.55
Triceps skinfold (mm)	17.00 (7.00)	11.00 (5.50)	<0.001	0.30
Subscapular skinfold (mm)	15.00 (8.00)	12.00 (5.00)	0.004	0.08
Iliac crest skinfold (mm)	20.00 (5.00)	14.38 (7.75)	<0.001	0.19
Abdominal skinfold (mm)	23.00 (7.00)	18.00 (10.00)	0.008	0.07
Waist circumference (cm)	74.10 (6.55)	78.05 (11.02)	<0.001	0.15
Hip circumference (cm)	95.10 (4.40)	96.00 (7.92)	0.930	0.00
Bi-styloid breadth (cm)	4.80 (0.30)	5.75 (1.40)	<0.001	0.46
Femur breadth (cm)	9.00 (0.90)	9.55 (0.80)	<0.001	0.20
Body fat percentage (%)	17.00 (4.70)	14.45 (4.05)	<0.001	0.17
Bone mass (kg)	8.50 (1.30)	12.00 (2.40)	<0.001	0.64
Residual mass (kg)	12.70 (2.10)	16.80 (4.13)	<0.001	0.48
Muscle mass (kg)	21.50 (5.60)	27.85 (7.02)	<0.001	0.31
BMI (kg/m^2^)	22.90 (2.70)	23.70 (2.95)	0.258	0.23
Waist-to-hip ratio (WHR)	0.77 (0.04)	0.83 (0.04)	<0.001	0.48
Minimum body mass (kg)	46.70 (4.30)	56.50 (5.67)	<0.001	0.55
Maximum body mass (kg)	62.90 (5.70)	75.80 (7.75)	<0.001	0.56
Age (years)	22.00 (5.00)	22.00 (4.00)	0.602	0.00

Note: Between-group comparisons were performed using the Mann–Whitney U test due to non-normal data distribution.

**Table 2:** Foot arch indices by author, sex, and foot (mean ± SD).

Female			Male	
Author/Index	Right	Left	Right	Left
Hernández-Corvo	65.01 ± 9.30	64.93 ± 7.02	62.31 ± 11.34	64.04 ± 8.23
Cavanagh	0.34 ± 0.04	0.34 ± 0.04	0.36 ± 0.04	0.36 ± 0.04
Chippaux–Smirak	33.32 ± 8.58	33.60 ± 6.56	35.85 ± 10.68	35.16 ± 8.17
Staheli index	57.86 ± 15.40	58.53 ± 12.34	63.42 ± 19.22	60.57 ± 14.58
Clarke’s Angle	50.28 ± 7.14	50.26 ± 7.04	41.82 ± 11.20	42.90 ± 9.97

**Table 3:** Significant Spearman’s Correlations between Foot Indices and Anthropometric Variables.

Foot Index	Associated Variable	Spearman’s Rho	*p*-Value
Left Foot—Cavanagh Arch Index	Iliac crest skinfold	−0.23	0.022
Left Foot—Cavanagh Arch Index	Hip circumference	−0.289	0.004
Left Foot—Cavanagh Arch Index	Bi-styloid breadth	0.214	0.033
Left Foot—Cavanagh Arch Index	Age	0.267	0.008
Right Foot—Clarke’s Angle	Body mass	−0.217	0.031
Right Foot—Clarke’s Angle	Bi-styloid breadth	−0.228	0.023
Right Foot—Clarke’s Angle	Femur breadth	−0.329	<0.001
Right Foot—Clarke’s Angle	Bone mass	−0.338	<0.001
Right Foot—Clarke’s Angle	Residual mass	−0.25	0.013
Right Foot—Clarke’s Angle	Muscle mass	−0.207	0.04
Right Foot—Clarke’s Angle	Waist-to-hip ratio (WHR)	−0.225	0.025
Left Foot—Clarke’s Angle	Body mass	−0.268	0.007
Left Foot—Clarke’s Angle	Height	−0.206	0.04
Left Foot—Clarke’s Angle	Bi-styloid breadth	−0.241	0.016
Left Foot—Clarke’s Angle	Femur breadth	−0.299	0.003
Left Foot—Clarke’s Angle	Bone mass	−0.375	<0.001
Left Foot—Clarke’s Angle	Residual mass	−0.266	0.008
Left Foot—Clarke’s Angle	Muscle mass	−0.249	0.013
Left Foot—Clarke’s Angle	Body mass index	−0.216	0.032
Left Foot—Clarke’s Angle	Maximum body mass	−0.225	0.025

**Table 4:** Association between sex and plantar foot type assessed by Clarke’s Angle.

Foot	χ^2^ (df = 4)	*p*-Value	Key Pattern by Sex
Right foot	24.10	<0.001	Higher prevalence of cavus and normal feet in females; higher prevalence of intermediate, flat, and planus-normal feet in males
Left foot	28.92	<0.001	Same sex-dependent distribution as the right foot, with a stronger differentiation

## Data Availability

The datasets used and/or analyzed during the current study are available from the corresponding author upon reasonable request.
